# Effects of Radix Astragali and Its Split Components on Gene Expression Profiles Related to Water Metabolism in Rats with the Dampness Stagnancy due to Spleen Deficiency Syndrome

**DOI:** 10.1155/2017/4946031

**Published:** 2017-05-21

**Authors:** Wen-Xiao Zhao, Ning Cui, Hai-Qiang Jiang, Xu-Ming Ji, Xiao-Chun Han, Bing-Bing Han, Tong Wang, Shi-Jun Wang

**Affiliations:** ^1^School of Nursing, Shandong University of Traditional Chinese Medicine, Jinan 250355, China; ^2^School of Traditional Chinese Medicine, Shandong University of Traditional Chinese Medicine, Jinan 250355, China; ^3^Key Laboratory of Traditional Chinese Medicine Basic Research, Shandong University of Traditional Chinese Medicine, Jinan 250355, China

## Abstract

Radix Astragali (RA) with slight sweet and warm property is a significant “qi tonifying” herb; it is indicated for the syndrome of dampness stagnancy due to spleen deficiency (DSSD). The purpose of this research was to explore effects of RA and its split components on gene expression profiles related to water metabolism in rats with the DSSD syndrome for identifying components representing property and flavor of RA. The results indicated that RA and its split components, especially polysaccharides component, significantly increased the body weight and the urine volume and decreased the water load index of model rats. Our data also indicated differentially expressed genes (DEGs) related to water metabolism involved secretion, ion transport, water homeostasis, regulation of body fluid levels, and water channel activity; the expression of AQP1, AQP3, AQP4, AQP5, AQP6, and AQP8 was improved; calcium, cAMP, MAPK, PPAR, AMPK, and PI3K-Akt signaling pathway may be related to water metabolism. In general, results indicate that RA and its split components could promote water metabolism in rats with the DSSD syndrome via regulating the expression of AQPs, which reflected sweet-warm properties of RA. Effects of the polysaccharides component are better than others.

## 1. Introduction

In traditional Chinese medicine (TCM), Chinese herbal medicines have four properties and five flavors, and various properties and flavors of herbs exert different effects. The action of a herbal medicine is always not one but many [1]. The property and flavor theory is a guideline for using the herbal medicines. The property of a Chinese medicinal herb affects material and energy metabolism. In addition, the material basis of the property and flavor of a herbal medicine can be split [2]. So far, however, there has been little scientific connotation of this theory based on the research, and we could not clearly explain its mechanism of property and flavor with modern medical theory. In this study, we tried to explore split components representing property and flavor of RA through studying effects of RA and its split components on gene expression profiles related to water metabolism in rats with the DSSD syndrome based on the property and flavor theory.

RA with slightly sweet flavor and warm property, known as Huang Qi in China, is one of the important “qi tonifying” or adaptogenic herbs. It possesses tonic, diuretic, and hepatoprotective properties and has been shown to exhibit immunomodulating, antioxidant, and antihyperglycemic effects [3–5]. The main components of RA include flavonoids, saponins, polysaccharides, amino acids, and some trance elements [4]. The China Pharmacopoeia recorded that RA affects the spleen and lung meridians and has the function of tonifying spleen qi [6]. It is indicated for symptoms of spleen qi deficiency such as fatigue, diarrhea, anemia, and loss of appetite [5].

Spleen governs dampness in the TCM theory. Spleen is the hub of water metabolism [7]. Water and cereal essence were digested, absorbed, and transported with the action of spleen qi. TCM holds the truth that the fat food, overfatigue, excessive thinking, and innate weakness of the body for a long time are the main causes of spleen qi deficiency. It has been confirmed that spleen qi deficiency will result in stagnancy of water and dampness [8, 9]. This is the pathogenesis of the DSSD syndrome. The clinical manifestations of DSSD include poor appetite, a bitter taste in the mouth, thick and slimy tongue, abdominal fullness, a feeling of discomfort, dizziness, weak or fatigued limbs, and frequently edema. Therefore, tonifying spleen qi and promoting diuresis are the main method for treating the DSSD syndrome. The DSSD syndrome is very common. Many chronic diseases such as obesity, ulcerative colitis, diabetes, and tumors may be accompanied with the DSSD syndrome, which will make disease recurrent and persistent [10–12]. However, there is no notion about the DSSD syndrome in modern medicine and there are no good treatments.

The effects of Chinese medicinal herbs on the syndrome involve multiple channels and targets because they contain complicated ingredients. The gene chip technology has a characteristic of high throughput, which can be used to analyze the complexity of herbs [13–16]. Therefore, in this study, we extracted the components of RA and observed effects of single components on water metabolism in rats with the DSSD syndrome to explore the mechanism and identify the bioactive components representing sweet-warm properties of RA using pharmacological experiment and gene chip technology.

## 2. Materials and Methods

### 2.1. Materials

RA (number 130107), the dried root of* Astragalus membranaceus* (Fisch.) Bge. var.* mongholicus* (Bge.) Hsiao, was purchased from Gansu province of China. Rat Gene Expression Microarray (8 × 60K, design ID: 028279), Gene Expression Hybridization Kit, the Quick Amp Labeling Kit, One-Color, Gene Chip Hybridization Oven, and Microarray Scanner were provided by Agilent Technologies Co., Ltd. (Shanghai, China); mirVana™ RNA Isolation Kit and 7500 Fast Dx Real-Time PCR Instrument were obtained from Applied Biosystems (Foster City, CA, USA); RNeasy Mini Kit was obtained from Qiagen Co., Ltd. (Hilden, Germany).

### 2.2. Animal

Fifty-six specific-pathogen-free (SPF), 4-week-old Wistar rats (half male and half female), weighing 150 ± 10 g, were purchased from Beijing Vital River Laboratory Animal Technology Co. Ltd. (Beijing, China). The animal certification number was SCXK (Jing) 2012-0001.

### 2.3. Animal Model

Rats were acclimated for 3 days in our animal facility and then randomly divided into 7 groups, a normal control group, a model group, a water decoction group, a flavonoids group, a saponins group, a polysaccharides group, and a residual aqueous solution group, with 8 rats in each group. Model rats with DSSD were induced according to the relevant reference [17]. Rats of the model group and the drug group were fed a special high-fat and low-protein diet improved from the AIN-76A purified rat diet and subjected to exhaustive swimming with tail load (10% of the body weight) every afternoon for 6 weeks. Rats were considered to be exhausted when their nose tip was submerged in water for 10 s. Rats of the normal control group were fed AIN-76A purified rat diet and distilled water.

### 2.4. Preparation of RA and Its Split Components

#### 2.4.1. Extracts of RA and Its Split Components

RA was decocted and concentrated to 0.54 g (crude drug)/mL. RA was treated through water boiling and precipitation with ethanol. The precipitate was polysaccharides. The supernatant was extracted by ethyl acetate and n-butyl alcohol. Flavonoids and saponins were obtained, respectively. Flavonoids contained calycosin glycosides, 4.88 mg·g^−1^, ononin, 3.80 mg·g^−1^, calycosin, 7.78 mg·g^−1^, and formononetin, 4.79 mg·g^−1^. Saponins contained astragaloside, 4.40 mg·g^−1^, astragalus saponin I, 0.28 mg·g^−1^, astragalus saponin II, 1.42 mg·g^−1^, and astragalus saponin III, 1.31 mg·g^−1^. Polysaccharides component was 402.2 mg·g^−1^ crude polysaccharides.

#### 2.4.2. Verification of Overlap among RA Split Components

The identification of overlap among RA split components was processed through similarity evaluation system for chromatographic fingerprint of TCM (Version 2012) with flavonoids as the control sample and principal component analysis using high-performance liquid chromatography-diode array detection (HPLC-DAD) data.

### 2.5. Experimental Protocol

After establishment of model rats, RA and its split components were administrated to rats. The dosage was determined from the product of highest daily dose (60 g/70 kg) of the Chinese Pharmacopoeia (2010 edition) and the equivalent dose coefficient. RA water decoction was given 5.40 g·kg^−1^·d^−1^. The dosage of the split components was the same as the content in the 5.40 g crude RA, such as the flavonoids component, 0.07 g·kg^−1^·d^−1^, the saponins component, 0.27 g·kg^−1^·d^−1^, the polysaccharides component, 1.41 g·kg^−1^·d^−1^, and the residual aqueous solution, 1.32 g·kg^−1^·d^−1^. The normal control group and the model group were given equal amounts of normal saline. Drugs were administered by gavage once a day for 14 days, 1 mL/100 g. After the last administration, rats were decapitated with their duodenum tissues separated at 4°C and put into liquefied nitrogen for storage.

### 2.6. Measurement of the Body Weight

After the last administration, the body weight of rats in each group was measured by electronic balance.

### 2.7. Measurement of the Water Load Index and the Urine Volume

The body weight of rats was detected after fasting for 12 hours, as a control value. Then rats were intraperitoneally injected with normal saline in the volume equal to 10% of the body weight. After that, rats were put into the metabolic cage, and the body weight was measured after 0, 1, 2, 4, and 6 hours. During this time, food and water were banned. Urine in 6 hours was collected. Ratio of the body weight loss (%) = (the body weight after water load − body weight before water load)/body weight before water load × 100. Water load chart between ratio of the body weight loss and time was drawn using origin 9.0; water load index was area under the curve of 6-hour water load.

### 2.8. Statistical Analysis

SPSS 21.0 was used for statistical analysis. The values for the body weight, water load index, and urine volume were all presented as the mean ± standard deviation (±SD). Differences between groups were tested by one-way ANOVA followed by LSD method to test for difference between the mean values of all groups. *P* < 0.05 was considered statistically significant. The area under the curve of 6-hour water load was calculated using Origin 9.0.

### 2.9. Gene Chip Analysis

Total RNA was extracted using mirVana RNA Isolation Kit and purified with RNeasy Mini Kit according to the manufacturers' instructions. Quality and concentration of RNA were checked by ND-2000 spectrophotometer. Equal amount of purified RNA from three different samples was mixed. Samples were hybridized separately into seven Agilent Rat Gene Expression Microarrays. 200 ng total RNA was used to synthesize double-stranded cDNA and then to produce biotin-tagged cRNA using the Quick Amp Labeling Kit, One-Color. The cRNA was purified by the RNeasy Mini Kit. The labeled cRNA samples were fragmented for 30 min at 60°C in the dark. The fragmented cRNA was hybridized to Agilent 8 × 60K Rat Gene Expression Microarray. Hybridization was performed using the Agilent Gene Expression Hybridization Kit at 65°C with rotation for 17 hours on an Agilent Gene Chip Hybridization Oven. The gene chips were washed followed by scanning with an Agilent Microarray Scanner. The images were converted into gene expression data by Agilent Feature Extraction software (version 10.7.1.1).

### 2.10. Functional and Pathway Enrichment Analysis of DEGs

Raw data obtained from gene chip test were imported into Agilent GeneSpring software (version 12.5) and normalized using quantile algorithm. The normalized values were compared between the model group and the normal control group and between treatment groups and the model group. The DEGs were screened using criterion of more than or less than 2-fold change. DAVID (https://david.ncifcrf.gov/) is an online program that provides a set of high-throughput gene functional annotation to understand the biological meaning behind plenty of genes [18]. To gain insight into the potential functional consequences and pathway changes induced by RA and its split components, GO and KEGG pathway enrichment analysis of DEGs were performed using DAVID database. The significant enrichment was defined as *P* < 0.05.

### 2.11. Quantitative Real-Time PCR Analysis

Two-step quantitative real-time PCR (qRT-PCR) was performed to confirm gene chip results according to Applied Biosystems' guidelines. Two candidate genes were chosen for qRT-PCR according to the expression ratio in each group. The primers of candidate genes were designed using Primer Premier 5.0 software. Sequences of oligonucleotide primer pairs for selected genes were shown in Table 1. Amplification conditions were 95°C for 30 s, followed by 40 cycles of 95°C for 3 s, 60°C for 30 s, and 60°C for 30 s. Melting curve analysis was performed at the end of each PCR reaction to confirm if a single PCR product was detected. The housekeeping gene, glyceraldehyde-3-phosphate dehydrogenase (GAPDH), was used to normalize quantification of the mRNA targets. All the reactions were performed in triplicate. The 2^−ΔΔCt^ method was used to calculate the relative gene expression levels.

## 3. Results

### 3.1. Identification of Overlap among RA Split Components

**Figure 1:**
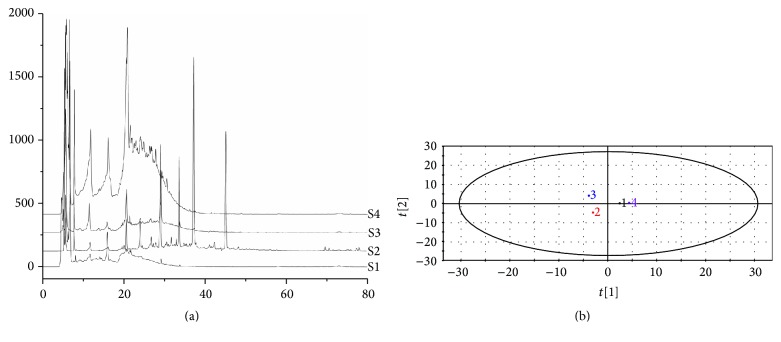
Verification of overlap among RA split components. (a) Fingerprint with flavonoids as control sample. (b) Scatter plot of principal components analysis using HPLC-DAD data. S1, 1: polysaccharides component; S2, 2: flavonoids component; S3, 3: saponins component; and S4, 4: residual aqueous solution.

Four main components, polysaccharides, flavonoids, saponins, and residual aqueous solution constituents, were extracted from RA. The results of fingerprint showed that overlap among the four components was very low, which totally meets the requirement for experimental standard (Figure 1(a)). HPLC-DAD data was imported into SIMCA-P to process multivariate statistical analysis. Two-dimensional distribution of four split components is shown in Figure 1(b), which also indicated that the overlap was very low.

### 3.2. Comparisons of the Body Weight

**Figure 2:**
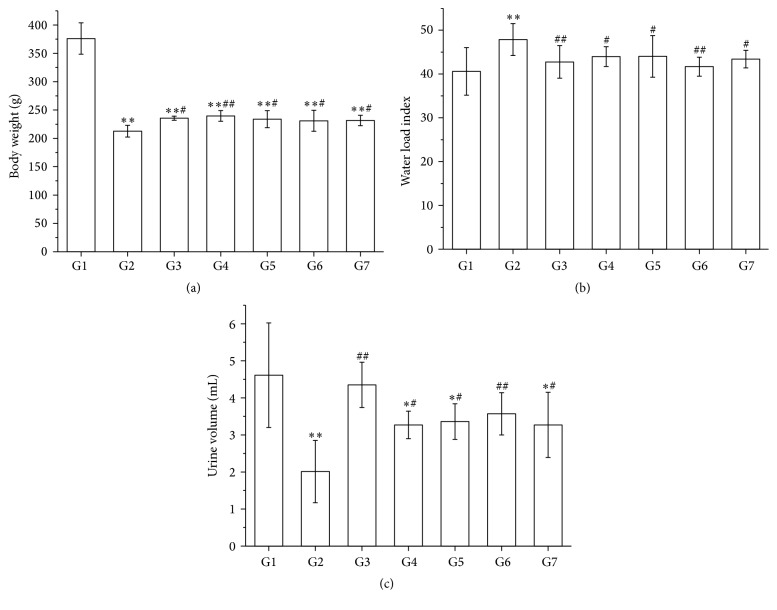
Effects of RA and its split components on the body weight, the water load index, and the urine volume of rats (a–c). G1: normal control group, G2: model group, G3: RA decoction group, G4: flavonoids group, G5: saponins group, G6: polysaccharides group, and G7: residual aqueous solution group. ^*∗*^*P* < 0.05 and ^*∗∗*^*P* < 0.01, compared with the normal control group; ^#^*P* < 0.05 and ^##^*P* < 0.01, compared with the model group; *n* = 8 in each group. Groups in this figure are the same as those in Table 2, Table 3, Table 4, and Figure 3.

Compared with the model group, the body weights of rats in the RA decoction group and split components groups were higher than that in the model group (*P* < 0.05 and *P* < 0.01, Figure 2(a)).

### 3.3. Comparisons of the Water Load Index and the Urine Volume

The water load index in RA and its split components groups was decreased, and the urine volume was increased compared with the model group (*P* < 0.05 and *P* < 0.01, Figures 2(b) and 2(c)).

### 3.4. Identification of DEGs

A total of 1275 DEGs were identified from datasets comparing the model group with the normal control group. Compared with the model group, the number of DEGs of the RA decoction group, the flavonoids group, the saponins group, the polysaccharides group, and the residual aqueous solution group was, respectively, 688, 1204, 1361, 3181, and 2419.

### 3.5. Functional Enrichment Analysis of DEGs and Genes Related to the Water Metabolism

To gain further insight into the function of DEGs, functional and pathway enrichment analysis was performed using DAVID. GO enrichment analysis' results showed that DEGs related to water metabolism in the model group were significantly enriched in secretion, ion transport, and regulation of body fluid levels (Table 2). Functional terms enriched by DEGs of RA decoction group were mainly involved in secretion, cellular homeostasis, and regulation of body fluid levels. The flavonoids group's DEGs were enriched in secretion, water homeostasis, and water channel activity, the saponins group's DEGs were enriched in ion transport, cellular water homeostasis, and water transport, the polysaccharides group's DEGs were enriched in ion transport, secretion, regulation of body fluid levels, and cellular homeostasis, and the residual aqueous solution group's DEGs were enriched in ion transport, secretion, and cellular homeostasis (Table 2). Studies reported that aquaporins (AQPs) were the most important molecular targets of the DSSD syndrome [10, 19]. As shown in Table 3, the study identified AQPs from DEGs including AQP1, AQP3, AQP4, AQP5, AQP6, and AQP8.

### 3.6. KEGG Pathway Enrichment Analysis of DEGs

Table 4 showed significantly enriched pathways of DEGs through KEGG enriched analysis. DEGs of the model group were enriched in neuroactive ligand-receptor interaction and calcium signaling pathway compared with the normal control group. Compared with the model group, the RA decoction group's DEGs were enriched in cytokine-cytokine receptor interaction, the flavonoids group's DEGs were enriched in cAMP and calcium signaling pathway, the saponins group's DEGs were enriched in PPAR signaling pathway, the polysaccharides group's DEGs were enriched in MAPK, cAMP, and PI3K-Akt signaling pathway, and the residual aqueous solution group's DEGs were enriched in PPAR, calcium, and AMPK signaling pathway.

### 3.7. Results of Quantitative Real-Time PCR

**Figure 3:**
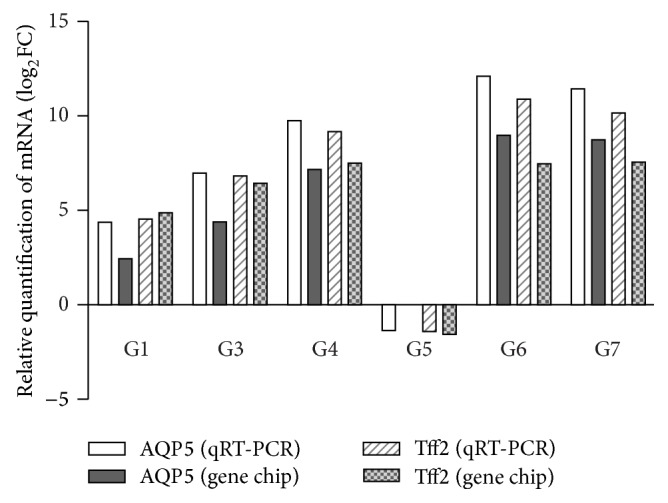
Fold change (FC) obtained using qRT-PCR revealed parallel patterns of gene expression found with gene chip analysis. Fold expression change with a positive sign indicates upregulation of the gene, whereas downregulation is represented by a negative sign. FC based on the results obtained with qRT-PCR or gene chip analysis. FC: relative gene expression level. log_2_⁡ FC is the vertical coordinate. All groups were compared with the model group.

To confirm gene chip results, qRT-PCR was performed on candidate genes and revealed parallel patterns of gene expression. As shown in Figure 3, the relative expression levels of AQP5 and Tff2 mRNA were in accord with the results of gene chip analysis.

## 4. Discussion

In this study, results showed that RA decoction and its split components increased the body weight of rats with the DSSD syndrome, decreased the water load index, and increased the urine volume in different degree, especially the polysaccharides component. The pharmacodynamic study indicated that RA and its split components could regulate the water metabolism of rats and have sweet-warm property.

The TCM theory emphasizes that spleen qi promotes water transport. The intestine is a main part regulating water absorption and secretion. Water transport across the intestine is important for maintaining body water homeostasis and ensures digestive and absorptive functions [20]. Therefore, the intestine is the main organ for studying the mechanism of DSSD. The importance of aquaporins (AQPs), transmembrane water channel proteins, in the gastrointestinal tract appears to be immediately evident [20]. The function of AQPs in the gastrointestinal gut may be involved in water transfer, secretion of water and fluid, and absorption of water and even small solutes through epithelium [21–23]. Until now, at least 11 AQPs (AQP1–11) have been found to be present in the whole gut [24]. Researches pointed that AQPs in the gastrointestinal tract are the molecular basis of the spleen governing dampness [19]. Studies also showed that the expression of AQPs changed in the gastrointestinal tract of rats with the DSSD syndrome, including AQP1, AQP2, AQP3, and AQP4 [20, 25, 26]. However, these researches were based upon a single gene or protein and have little mechanism study.

In the present study, we screened out DEGs of the duodenum using the gene chip technology and performed functional and KEGG pathway enrichment analysis. Table 3 showed that GO terms related to water metabolism contained secretion, ion transport, regulation of body fluid levels, water homeostasis, and water transport. Results of the GO enrichment analysis indicated that water metabolism in the body with the DSSD syndrome was disordered. The results showed that the expression of AQP1, AQP3, AQP4, AQP5, AQP6, and AQP8 was changed in model rats; RA and its split components could reverse these changes. AQP1 is widely distributed at the capillary endothelium of the mucosa and submucosa, and that indicates a main role of AQP1 in the passage of water between the gastrointestinal mucosa and bloodstream [24, 27, 28]. AQP1 is also located in endothelial cells of central lacteals in the villi of the small intestine [29]. AQP3 is expressed at the basolateral membrane of epithelial cells at villous tip and could mediate permeation of glycerol and some small neutral solutes as well as water; it serves in water reabsorption [30]. AQP4 is present in the basolateral membrane of the crypt cells located at the bottom of the crypt [31]. In addition to AQP5 expression, AQP5 is located at the apical and lateral membranes of Brunner's gland in the duodenum, where it involves a water secretion mechanism [32–34]. Earlier studies have demonstrated that AQP6 is present along the rat small intestine, in addition to direct involvement in the absorption of water and anions [35]. AQP8 is present in absorptive epithelial cells but it does not play a major role in intestinal transcellular water transfer. In the intestine, water metabolism may involve water and fluid secretion and absorption. Therefore, the results suggested that RA and its split components could change the expression of AQPs mediated water secretion or absorption of the duodenum to improve dampness stagnancy. The split components had the most influence on AQP5 expression, especially the polysaccharides component.

The secretion and absorption of transepithelial fluids are closely related to intestine physiology by maintaining water and electrolyte balance [24, 35]. The balance of intestine fluid movement has been reported to be regulated by complicated factors, including gut hormones, intestinal contents, inflammatory factors, and feed conditions [33]. These regulations may affect alterations of AQPs expression and distribution, which may account for the changes of water transport in the gut [22, 36]. In this study, KEGG pathway analysis indicated that the regulatory mechanism of water transport may be involved in MAPK, PPAR, cAMP, PI3K-Akt, and calcium signaling pathway. KEGG enrichment analysis also showed that DEGs were enriched in cytokine-cytokine receptor interaction in the model group, the saponins group, and the polysaccharides group and neuroactive ligand-receptor interaction in the residual aqueous solution group. Although these pathway enrichment *P* values were not significant, they had an important meaning in the biological function. It indicated that RA and its split components produced efficacy through multitargets and multipathways. Therefore, we inferred that RA and its split components regulated the level of the cytokine, gastrointestinal hormone, which triggered MAPK, PPAR, cAMP, calcium, and PI3K-Akt signaling pathway and then activated downstream molecules, finally regulating transcription of a series of target genes including AQPs.

In conclusion, RA and its split components could regulate AQPs via multipathway to promote water metabolism of rats with the DSSD syndrome, which indicated the sweet-warm properties of RA. The polysaccharides component had better effects among the split components. Further study was needed for the detail of the regulative mechanism.

## Figures and Tables

**Table 1:** The primers of selected genes for qRT-PCR.

GenBank accession number	Gene symbol	Forward primer (5′-3′)	Reverse primer (5′-3′)
NM_012779	AQP5	CACACCAGAAAGGGACGACA	TCCGTGAGCCATCTATCCCT
NM_053844	Tff2	GATCTTCGAAGTGCCCTGGT	ACCCCAGAGATCGACACAGT
NM_017008	GAPDH	CAGGGCTGCCTTCTCTTGTG	TGGTGATGGGTTTCCCGTTG

**Table 2:** Functional enrichment analysis of DEGs related to water metabolism.

Group	Term	Description	Count	*P* value
G2 versus G1	GO:0046903	Secretion	55	5.54*E* − 04
	GO:0006811	Ion transport	65	1.62*E* − 03
	GO:0019725	Cellular homeostasis	41	7.85*E* − 03
	GO:0050878	Regulation of body fluid levels	20	1.92*E* − 02

G3 versus G2	GO:0046903	Secretion	40	5.93*E* − 05
	GO:0019725	Cellular homeostasis	29	2.62*E* − 03
	GO:0006811	Ion transport	38	1.92*E* − 02
	GO:0050878	Regulation of body fluid levels	13	3.64*E* − 02

G4 versus G2	GO:0046903	Secretion	72	1.83*E* − 07
	GO:0006811	Ion transport	85	5.64*E* − 07
	GO:0051046	Regulation of secretion	54	8.29*E* − 07
	GO:0048878	Chemical homeostasis	66	1.35*E* − 05
	GO:0050878	Regulation of body fluid levels	30	2.36*E* − 05
	GO:0030104	Water homeostasis	8	2.94*E* − 03
	GO:0042044	Fluid transport	5	1.47*E* − 02
	GO:0015250	Water channel activity	4	1.02*E* − 02

G5 versus G2	GO:0006811	Ion transport	82	2.64*E* − 05
	GO:0009992	Cellular water homeostasis	4	1.03*E* − 02
	GO:0042044	Fluid transport	5	1.75*E* − 02
	GO:0006833	Water transport	4	3.40*E* − 02
	GO:0015250	Water channel activity	4	1.27*E* − 02

G6 versus G2	GO:0006811	Ion transport	208	1.53*E* − 09
	GO:0046903	Secretion	154	3.76*E* − 06
	GO:0050878	Regulation of body fluid levels	59	1.33*E* − 04
	GO:0019725	Cellular homeostasis	117	2.65*E* − 04
	GO:0006884	Cell volume homeostasis	8	1.61*E* − 02
	GO:0007589	Body fluid secretion	19	3.35*E* − 02
	GO:0005216	Ion channel activity	48	3.78*E* − 02

G7 versus G2	GO:0006811	Ion transport	183	5.84*E* − 12
	GO:0046903	Secretion	139	1.00*E* − 08
	GO:0050878	Regulation of body fluid levels	56	1.51*E* − 06
	GO:0019725	Cellular homeostasis	104	8.78*E* − 06
	GO:0030104	Water homeostasis	13	1.09*E* − 03
	GO:0015267	Channel activity	50	3.24*E* − 03
	GO:0050891	Multicellular organismal water homeostasis	9	1.92*E* − 02

**Table 3:** Expression of AQPs in each group.

GenBank accession number	Gene symbol	FC (G2 versus G1)	FC (G3 versus G2)	FC (G4 versus G2)	FC (G5 versus G2)	FC (G6 versus G2)	FC (G7 versus G2)
NM_012778	AQP1	—	—	2.08	—	4.90	4.53
NM_031703	AQP3	—	—	—	−2.95	—	—
NM_001142366	AQP4	—	—	—	3.01	—	—
NM_012779	AQP5	−5.41	21.06	144.07	—	504.90	429.01
NM_022181	AQP6	—	—	2.03	—	—	—
NM_019158	AQP8	—	−2.19	—	−6.14	−2.07	−6.30

Note: FC: fold change; —: no changes of gene expression.

**Table 4:** KEGG pathway enrichment analysis of DEGs.

Group	Term	Description	Count	*P* value	Genes
G2 versus G1	KEGG:rno04080	Neuroactive ligand-receptor interaction	20	2.10*E* − 03	GPR83, CCKAR, TAAR7B, CCKBR, OPRL1, LEPR, OXTR, GRM1, CRHR2, ADRB1, NMUR1, GALR3, HTR6, GRM6, UTS2R, TAAR5, LHB, GLP1R, MTNR1A, HTR5B
	KEGG:rno04020	Calcium signaling pathway	12	3.16*E* − 02	CCKAR, SLC8A2, ADRB1, CCKBR, CALML3, HTR6, SPHK1, CACNA1G, OXTR, CAMK2A, GRM1, HTR5B

G3 versus G2	KEGG:rno04060	Cytokine-cytokine receptor interaction	11	1.14*E* − 02	VEGFB, IL1R2, CCL20, CXCL13, TNFRSF12A, IL18, CCR2, IL13, CSF3R, IL6R, TNFSF9

G4 versus G2	KEGG:rno04024	cAMP signaling pathway	17	8.27*E* − 04	ADCY3, VAV3, ADCY2, GRIN1, ATP1A3, CREB5, VAV2, ATP2B2, FOS, ATP2B3, SSTR2, CREB3L4, RAPGEF3, ADCY10, LIPE, GLP1R, AKT2
	KEGG:rno04020	Calcium signaling pathway	16	1.35*E* − 03	ADCY3, CCKAR, GNA15, ADCY2, TACR3, CCKBR, GRIN1, SPHK1, BDKRB1, NTSR1, ATP2B2, ATP2B3, PLCD4, CACNA1E, HTR2B, CACNA1A
	KEGG:rno04152	AMPK signaling pathway	11	1.01*E* − 02	SLC2A4, LEPR, SCD, PFKFB1, CREB3L4, CREB5, ADIPOQ, IRS1, LIPE, AKT2, PCK1
	KEGG:rno04080	Neuroactive ligand-receptor interaction	17	3.62*E* − 02	CCKAR, TAAR7B, TACR3, CCKBR, GRIK2, LEPR, GRIN1, PRSS1, BDKRB1, NTSR1, SSTR2, GALR1, LOC286960, PRSS3, HTR2B, GLP1R, GABRP

G5 versus G2	KEGG:rno03320	PPAR signaling pathway	10	3.80*E* − 03	CPT1B, APOA2, ACADM, PPARG, AQP7, GK, FABP2, ADIPOQ, SLC27A2, ACSL6

G6 versus G2	KEGG:rno04010	MAPK signaling pathway	40	6.60*E* − 04	IL1R2, IL1R1, FGF7, FGFR3, TNF, PDGFB, MAP4K2, CACNB1, FGF13, HSPA1B, CACNB4, TGFB2, KRAS, RAC2, SOS2, FAS, IL1A, HSPA8, AKT2, PRKCA, PTPN5, TAOK3, PTPRR, NR4A1, FGF20, MECOM, STK4, RPS6KA5, PLA2G4A, DUSP2, MAPK12, JUN, MAPK8, CACNA1E, MAPK8IP1, GADD45B, MAP3K14, GADD45A, DUSP8, CD14
	KEGG:rno04024	cAMP signaling pathway	28	1.29*E* − 02	PPARA, ADCY4, ADCY8, CNGB1, GRIN3A, SOX9, GLI3, ATP2B1, ATP2B2, ATP2B3, RAC2, CREB3L1, CREB3L4, CAMK2B, RAPGEF3, ADCY10, AKT2, PLD1, VAV3, ROCK2, ADCY9, JUN, ABCC4, GNAS, MAPK8, GHSR, GLP1R, LIPE
	KEGG:rno04151	PI3K-Akt signaling pathway	41	3.65*E* − 02	FGF7, PHLPP2, FGFR3, PDGFB, GNG13, KITLG, VTN, FGF13, BCL2L1, IGF1R, LAMB3, KRAS, COMP, ATF6B, TEK, SOS2, CREB3L1, GYS2, CREB3L4, THBS1, PPP2R2B, COL11A1, ANGPT2, AKT2, GHR, PRKCA, SGK1, SGK2, IL7, ITGA2, NR4A1, IL6R, FGF20, VEGFB, LAMA2, LAMA1, VEGFA, GNB5, LAMC2, LAMC1, INS1

G7 versus G2	KEGG:rno03320	PPAR signaling pathway	13	1.11*E* − 02	LPL, CYP4A1, CYP4A3, SCD, ADIPOQ, RGD1565355, PCK1, APOA2, CD36, LOC681458, APOC3, CYP4A8, FABP5
	KEGG:rno04020	Calcium signaling pathway	23	1.95*E* − 02	ORAI2, GNA15, CCKBR, ADCY8, TACR2, TACR1, SPHK1, BDKRB1, ATP2B2, P2RX7, CHRM5, PLCB3, ATP2B3, CAMK4, ADCY9, ATP2A3, P2RX2, TBXA2R, PLCD4, CHRNA7, HTR2B, PLCB2, CACNA1A
	KEGG:rno04152	AMPK signaling pathway	17	2.65*E* − 02	LEPR, SCD, IRS1, ADIPOQ, RGD1565355, PCK1, IGF1R, G6PC, EIF4EBP1, CD36, LOC681458, CREB3L1, GYS2, CREB3L4, PIK3R3, PPP2R2B, LIPE
